# Sexual Dimorphism of Pain Control: Analgesic Effects of Pioglitazone and Azithromycin in Chronic Spinal Cord Injury

**DOI:** 10.1089/neu.2018.6207

**Published:** 2019-07-12

**Authors:** John C. Gensel, Renée R. Donahue, William M. Bailey, Bradley K. Taylor

**Affiliations:** ^1^ Spinal Cord and Brain Injury Research Center and Department of Physiology, University of Kentucky College of Medicine, Lexington, Kentucky.; ^2^ Department of Anesthesia and Perioperative Medicine, the Pittsburgh Center for Pain Research, and the Opioid Research Center at the University of Pittsburgh, University of Pittsburgh School of Medicine, Pittsburgh, Pennsylvania.

**Keywords:** macrophage, microglia, PPAR, sex, thiazolidinedione

## Abstract

Central neuropathic pain develops in greater than 75% of individuals suffering a spinal cord injury (SCI). Increasingly, sex is recognized as an important biological variable in the development and treatment of peripheral neuropathic pain, but much less is known about the role of sex in central neuropathic pain and its pharmacological inhibition. To test the hypothesis that efficacy of analgesic therapies differs between males and females in SCI, we used a mouse model of SCI pain to determine the analgesic efficacy of pioglitazone (PIO), U.S. Food and Drug Administration–approved drug for the treatment of diabetes, and azithromycin (AZM), a commonly prescribed macrolide antibiotic with immunomodulatory properties. Male and female mice received moderate-severe T9 contusion SCI (75-kdyn). A robust heat hyperalgesia developed similarly between male and female mice by 4 weeks post-injury and lasted throughout the duration of the study (14 weeks). Three months after SCI, mice were treated with PIO (10 mg/kg, intraperitoneal) or AZM (160 mg/kg, oral). We observed a sex-specific effect of PIO with significant antihyperalgesic effects in females, but not males. In contrast, AZM was effective in both sexes. Our data support the use of PIO and AZM as novel therapies for SCI pain and highlight the importance of considering sex as a biological variable in clinical and experimental SCI pain research.

## Introduction

Central neuropathic pain develops in greater than 75% of individuals suffering a spinal cord injury (SCI).^1^ U.S. Food and Drug Administration (FDA)-approved analgesic drugs for SCI pain are only partially effective in a subset of patients.^2,3^ One potential confound in the pharmacological management of SCI pain could be sexual dimorphism. Although the magnitude and frequency of overt pain responses are similar between males and females in models of peripheral neuropathic pain, the ability of some analgesics to inhibit pain is sexually dimorphic.^4–7^ Whether this extends to the central neuropathic pain of SCI, however, is unclear because the great majority of pre-clinical studies do not consider sex as a potential factor that might influence therapeutic efficacy.^8^ To begin to address this question, we evaluated the ability of two FDA-approved drugs to reduce pain in male and female SCI mice: pioglitazone (PIO), an insulin sensitizer shown to reduce hyperalgesia in models of spinal cord or nerve injury,^9–13^ and azithromycin (AZM), an antibiotic that limits SCI macrophage activation, a purported driver of SCI pain.^14–17^

## Methods

### Animals

Experiments were performed using 3- to 4-month-old male and female C57BL/6 mice (The Jackson Laboratory, Bar Harbor, Maine). Animals were housed in IVC cages with *ad libitum* access to food and water. All procedures were performed in accord with the guidelines of the Office of Responsible Research Practices and with the approval of the Institutional Animal Care and Use Committee at the University of Kentucky.

### Spinal cord injury

A 75-kdyn, T9, mid-thoracic contusion SCI was produced using the Infinite Horizons (IH) injury device (Precision Systems and Instrumentation, Fairfax Station, VA)^18^ as described in detail previously.^14^ Briefly, a T9 laminectomy was performed under intraperitoneal (i.p.)-delivered anesthetic (ketamine [100 mg/kg] and xylazine [10 mg/kg]). Animals then received SCI with the IH device. Spinal cord displacement values (T = 1.4; *df* = 12; *p* = 0.20; male, 704.7 ± 42.7 μm; female, 635.8 ± 28.5 μm) and impact force values (T = 0.28; *df* = 12; *p* = 0.78; male, 77.8 ± 1.0 kdyn; female, 78.13 ± 0.48 kydn) were not significantly different between sexes, ensuring that injury biomechanics did not differ between males and females. After surgery, animals received one subcutaneous injection of buprenorphine-SR (1 mg/kg) for pain and 2 mL of saline + antibiotic (5 mg/kg, enrofloxacin 2.27%; Norbook Inc, Lenexa, KS) and then were housed in warming cages overnight. SCI animals continued to receive 1 mL of saline + prophylactic enrofloxacin subcutaneously daily for 5 days. Bladder expression was performed on injured mice twice-daily until mice reached voluntary evacuation.

### Heat hyperalgesia

Heat hyperalgesia was assessed using a unilateral hindpaw withdrawal test as described previously.^9,19^ Animals were first acclimated to the testing apparatus (glass surface within an acrylic enclosure). The plantar surface of each hindpaw was individually exposed to an infrared intensity of 25 units, and the latency to a unilateral hindpaw withdrawal response (e.g., jumping, licking, and flicking) was automatically recorded. The animal was immediately removed after the withdrawal response or at a cutoff of 30 sec to avoid tissue injury. Average latency of both hindpaws is reported for each time point.

### Experimental design

Investigators blinded to experimental groups performed all data acquisition and analyses. Data from 15 mice (8 females and 7 males) are reported in the current study. One male was excluded from the study based upon *a priori* exclusion criteria of an abnormal time versus force curve (indicative of a bone hit at the time of SCI). A second male died at the time of injury (the impact curve indicated spinal cord movement with excessive displacement) and was immediately replaced. Within-subject crossover studies were utilized to evaluate the effect of pioglitazone (initiated at 10 weeks post injury with a 1-week washout) or azithromycin (initiated at 13 weeks post-injury with a 1-week washout). Order of treatment was randomized for each subject within balanced treatment groups. PIO (10 mg/kg, i.p.; Cayman Chemical, Ann Arbor, MI) or vehicle (saline) was administered at a 10-mL/kg volume after development of chronic heat hypersensitivity (10 or 11 weeks post-SCI). Next, at 13 weeks post-injury, we administered AZM (160 mg/kg, oral gavage [o.g.], generated by crushing Zithromax tablets and suspending in 1% methylcellulose) or vehicle (1% methylcellulose) in a 0.1-mL volume. We chose a 10-mg/kg PIO dose based upon its effectiveness in reducing mechanical hypersensitivity in male rats in a spared nerve injury model of pain.^11^ The 160-mg/kg AZM dose used in the current study reduces intraspinal inflammation and improves recovery in female mice after SCI.^14,15^ For both studies, thermal hyperalgesia was measured every 30 min until antihyperalgesic effects of PIO or AZM had resolved.

### Statistical analysis

Statistical analyses were completed using GraphPad Prism software (version 7.0; GraphPad Software Inc., La Jolla, CA). Withdrawal responses were analyzed using two-way ANOVA (sex × time [repeated] or treatment × time [repeated]) followed by Holm-Sidak's test for multiple comparisons. F-values are reported for repeated measures. An independent sample t-test was used to compare spinal cord injury displacement between sexes. Results were considered statistically significant at *p* ≤ 0.05. All data are presented as mean ± SEM unless otherwise noted. Figures were prepared using Adobe Photoshop CS6 (Adobe Systems, San Jose, CA) and Prism software (version 7.0; GraphPad Software Inc.).

## Results

**FIG. 1.**
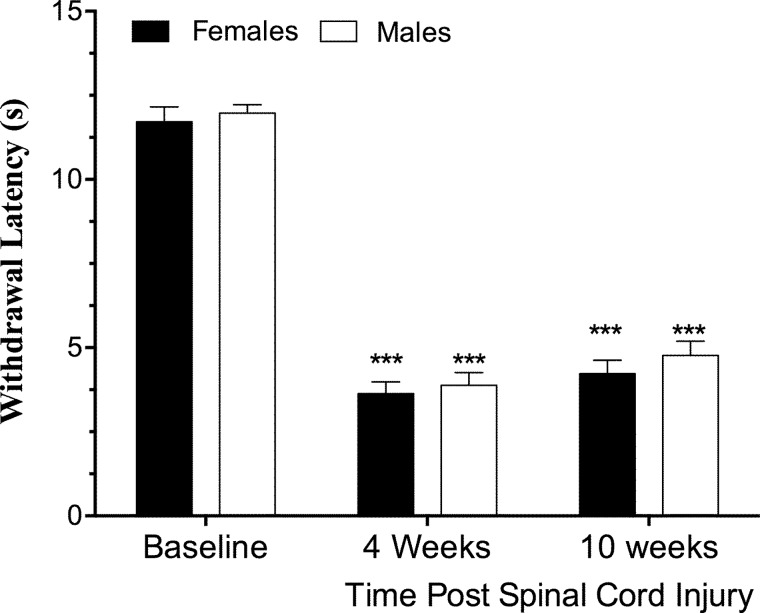
Sex does not alter the development of thermal hyperalgesia in mice after spinal cord injury (SCI). Adult (4-month-old) male and female mice received a moderate-severe thoracic contusion SCI (75-kdyn Infinite Horizons [Precision Systems and Instrumentation, Fairfax Station, VA] T9 contusion). Withdrawal latency to heat stimuli decreases after SCI, relative to baseline, indicative of hyperalgesia in both male and female mice. ***p < 0.0001 versus baseline, Holmes-Sidak post-hoc after repeated-measures analysis of variance (*p* < 0.0001, main effect of time). There were no differences between sexes (*p* > 0.2 main effect of sex; *p* > 0.9 sex × time interaction). *n* = 7–8, mean ± standard error of the mean.

When compared to pre-surgical baseline values, SCI reduced heat withdrawal latency at 4 and 10 weeks post-injury in both male and female mice (main effect of time, *F*_(2,26)_ = 231.6; *p* < 0.0001), with no significant difference between sexes (main effect of sex, *F*_(1,13)_ = 1.8; *p* = 0.20; sex × time interaction, *F*_(2,26)_ = 0.08; *p* = 0.92; *n* = 7–8; Fig. 1).

**FIG. 2.**
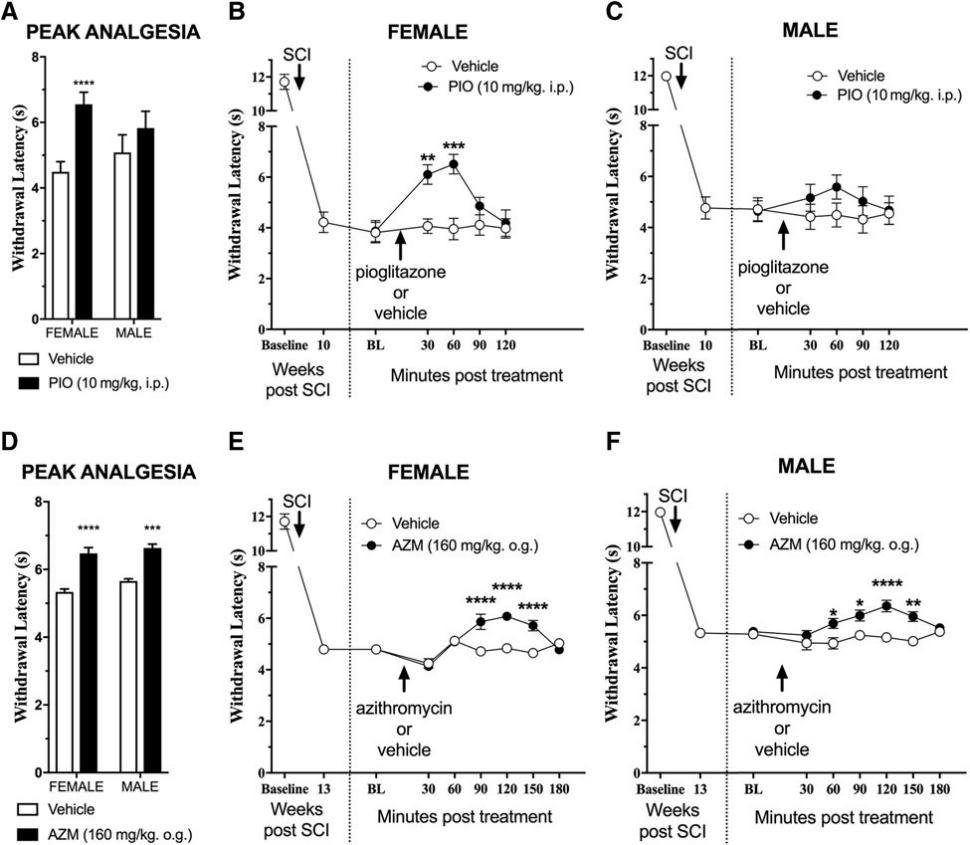
Pioglitazone (PIO) and azithromycin (AZM) alleviate spinal cord injury (SCI) pain in a sex-specific manner. Aged-matched (4-month-old) male and female mice received T9 75-kydn SCI. (**A**–**C**) A single intraperitoneal (i.p.) injection of PIO (10 mg/kg) was administered after the development of chronic pain (11 weeks post-SCI). (A) Analysis of the peak analgesia response (maximal withdrawal latency) revealed a significant sex × treatment interaction (*p* = 0.018) with significant improvements in female mice. (B) Treatment was only effective in female SCI mice (main effect of treatment, *p* < 0.05) versus male SCI mice (main effect of treatment, *p* = 0.4). PIO treatment significantly reduced pain responses in females at 30 and 60 min post-treatment. (**D**–**F**) A single dose of AZM (160 mg/kg) delivered by oral gavage (o.g.) was administered after the re-establishment of thermal hyperalgesia (2 weeks after PIO experiment in A; 13 weeks post-SCI). (D) Analysis of the maximal withdrawal latency recorded revealed no significant sex × treatment interaction (*p* = 0.49) with significant improvements in both female and male mice with AZM. (E and F) Treatment was effective in both female and male SCI mice (main effect of treatment by sex, *p* = 0.0008 and 0.0001, respectively). AZM treatment significantly reduced pain responses in females starting at 90 min post-treatment and in males at 60 min post-treatment (*p < 0.05; **p < 0.01; ***p < 0.001; ****p < 0.0001, Holmes-Sidak post-hoc after repeated-measures analysis of variance). Mean ± standard error of the mean, *n* = 7–8. BL, baseline.

At 10 weeks post-injury, we administered PIO (10 mg/kg, i.p.) and evaluated thermal hyperalgesia every 30 min until withdrawal responses returned to pre-treatment values (120 min total). To test the effect of sex on drug antihyperalgesic efficacy, we compared withdrawal latency at the peak analgesic effect for each animal under vehicle or drug treatment conditions to perform a two-way analysis of variance comparing withdrawal latency as a function of sex (male or female) and treatment (vehicle or PIO). This revealed a significant sex × treatment interaction for PIO (*F*_(1,13)_ = 7.3; *p* = 0.018). As illustrated in Figure 2A, subsequent analysis of each sex revealed that PIO reduced pain hypersensitivity in females, as indicated by increased withdrawal latencies (main effect of treatment, *F*_(1,14)_ = 5.2; *p* = 0.038). By contrast, PIO did not change withdrawal latencies in males (main effect of treatment, *F*_(1,12)_ = 0.67; *p* = 0.44).

Analysis of withdrawal latency at the peak analgesic effect of AZM or vehicle treatment for each animal did not yield a significant sex × treatment interaction (*F*_(1,13)_ = 0.5; *p* = 0.49). Subsequent analysis of each sex revealed that AZM significantly increased withdrawal latencies, both in females (main effect of treatment, *F*_(1,16)_ = 16.8; *p* = 0.0008) and males (main effect of treatment, *F*_(1,10)_ = 37.6; *p* = 0.0001).

## Discussion

There is growing experimental evidence that sex is an important biological variable in the pathophysiology of neurotrauma,^20,21^ and that development of neuropathic pain may be differentially regulated between males and females.^7^ We report that heat hyperalgesia develops similarly in male and female mice after SCI. This is consistent with recent observations reported in rats.^19^ Whether our results extend to other pain modalities, however, is unclear. For example, mechanical allodynia appears to be sexually dimorphic after a combined contusion/compression or photochemical SCI in rats.^19,22^ Pain hypersensitivity at the level of contusion SCI, as opposed to distal pain examined in the current article, is also sexually dimorphic in SCI rats.^23^ Further studies are needed to determine the role of SCI biomechanics, sensory modality, and methods of behavioral testing in the determination of sexual dimorphism in SCI pain.

Increasing evidence implicates sex to be an important biological determinant of analgesic efficacy.^7^ Clinically, prevalence of opiate and non-steroidal anti-inflammatory drug use is higher in woman than men with SCI who suffer with pain.^24^ However, fewer than 4% of published animal studies evaluating SCI pain include both males and females,^8^ and the effect of sex on analgesic efficacy is understudied in SCI. We report that both PIO and AZM effectively reduce, but do not eliminate, behavioral signs of SCI pain. These data highlight both drugs as potential analgesic therapies for SCI pain. Importantly, we found sex to be a critical determinant of therapeutic efficacy. We observed that PIO exerted a sexually dimorphic hyperalgesic effect because it increased withdrawal threshold in female, but not male, SCI mice. In contrast, AZM attenuated heat hypersensitivity in both sexes. These results highlight the idea that sex differences can obscure the effectiveness of pharmacotherapeutics for neuropathic pain and are critical when evaluating new SCI treatments.

One limitation of this study is the absence of sham controls. An ongoing study (manuscript in preparation) in male and female SCI mice indicates no effect of sex on pain responses in sham-injured controls (data not shown). A second limitation of this study is the application of a single dose of each drug. Indeed, although PIO was ineffective in males and only partially effective in females (it reduced, but did not eliminate, heat hypersensitivity), this does not necessarily preclude its effectiveness as a powerful analgesic after SCI. Perhaps higher doses may reveal analgesic effects for both sexes. Indeed, ongoing preliminary studies in a peripheral nerve injury model of neuropathic pain indicate that higher doses of pioglitazone are effective in both males and females, albeit with 100-fold greater potency in females (data not shown). Alternatively, other routes of administration (intravenous, intrathecal, or supraspinal) may efficiently target the site of action while minimizing first-pass metabolism. Synergistic approaches that combine PIO or AZM with other analgesics may also increase the therapeutic window. Thus, additional dose-response studies are needed to identify the floor and ceiling effects of these types of drugs and determine their translational potential in males and females after SCI.

Our results add to a growing body of literature supporting the therapeutic properties of PIO and other thiazolidinedione drugs for SCI.^12,25,26^ For example, Park and colleagues^12^ reported that PIO reduces SCI pain when delivered soon after SCI (sex not specified). Here, we observed analgesic effects when delivered months after SCI, a time course that mimics our previous studies of PIO for peripheral neuropathic pain that follows nerve injury.^9,10^ So, in contrast to previous studies that were restricted to early time points, the extended time course in our study generates greater impact in its translational relevance to chronic pain. Potential mechanisms of analgesic action of PIO include activation of peroxisome proliferator-activated receptor gamma, perhaps located on immune/inflammatory cells or glial cells, and maintenance of mitochondrial bioenergetics.^10–12,25^ Whether these mechanisms hold true in both sexes will require further studies.

AZM exerts immunomodulatory, neuroprotective properties in central nervous system trauma,^14,15,27^ and we extend the list of beneficial effects to analgesia, thus further supporting its use as a SCI therapy. AZM is the mostly commonly prescribed antibiotic for treating infections in SCI individuals^28^ and therefore has high translational potential in this patient population. The immunomodulatory effects of AZM are likely to be independent of antimicrobial activity.^29^ In SCI, stroke, and myocardial infarction, shifts in macrophage phenotype are concurrent with AZM-mediated tissue protection.^14,15,27,30^ Microglia and macrophage activation contribute to development and maintenance of neuropathic pain after SCI, and interventions that reduce glial activation reduce behavioral signs of chronic pain in animal models.^16,17^

In conclusion, we identified two novel therapies for treatment of established hyperalgesia after SCI. We observed that female, but not male, mice benefit from PIO treatment whereas AZM is effective in both sexes. Both therapies are FDA approved for human use, and AZM is commonly prescribed to SCI individuals. Recent findings of sexual dimorphism in neuropathic pain responses, along with increased focus on sex as a biological variable in basic and clinical research, highlight the importance of considering sex-specific differences in SCI pain responses. The results of the current work highlight the importance of including both sexes in pre-clinical analgesic studies.
